# The value conflict between freedom and security: Explaining the variation of COVID-19 policies in democracies and autocracies

**DOI:** 10.1371/journal.pone.0274270

**Published:** 2022-09-09

**Authors:** Nicole J. Saam, Carmen Friedrich, Henriette Engelhardt

**Affiliations:** 1 Department of Social Sciences and Philosophy, Friedrich-Alexander-University Erlangen-Nuremberg, Erlangen, Germany; 2 Department of Sociology, University of Bamberg, Bamberg, Germany; 3 The State Institute for Family Research (ifb), Bamberg, Germany; University of Catania, ITALY

## Abstract

In the name of health security, individual freedoms were constrained in an unprecedented way in many countries, democratic or authoritarian, all over the world during the COVID-19 pandemic. Yet the constraints have not been consistent across countries, which motivates this paper to examine the relevance of value preferences towards freedom or security in the society for COVID-19 policies. Based on data for 40 democratic and authoritarian countries, the analyses show that the variation in the stringency of COVID-19 policies can be explained by value preferences of the population only in autocracies. In democracies, however, we do not find such a relationship. Governments in democratic political systems, we argue, are responsive to their constitutions and face prosecution by the judiciary if they violate the law or provisions of the constitution, limiting their capacity to implement strong COVID-19 policies. Nevertheless, their COVID-19 policies restricted citizens’ freedoms and liberties, which means that these policies were rather not responsive to citizens’ preferences for freedom, democratic rights and liberties. By highlighting how autocracies respond to their citizens’ value preferences for security, this paper contributes to a better understanding of how autocracies might gain legitimacy in times of crises.

## 1 Introduction

In the name of health security, individual freedoms were constrained in an unprecedented way in many countries, democratic or authoritarian, all over the world during the COVID-19 pandemic. Nation-wide lockdowns have become standard policies in fighting the spread of COVID-19. Yet the constraints have not been consistent across countries, which have motivated researchers to explain variations across jurisdictions [1–3]. Studies on policies in democracies find that the constitutional protection of individual liberties had a negative effect on restrictions of freedoms [4]. Studies involving democracies and autocracies offer a mixed picture: Some find that democracies were slower in adapting COVID-19 policies [5, 6], while others find that democracy is not significant in predicting the speed of government stringency [7]. Frey et al. [8] show that autocracies have been stricter in their mobility restriction and contact tracing policies. Lins et al. [9], however, find no differences in social distancing policies between autocracies and democracies, and the level of democracy was not associated with the number of tests for COVID-19 [10]. Two studies include the overall freedom of the country: Toshkov et al. [3] find that a higher Freedom House global freedom score was related to a slower policy reaction in European countries, while Gutkowski [11] find that there was no relationship between lockdown timing and the degree of freedom in a sample of 128 countries. Toshkov et al. [3] conclude that “countries with higher freedom might have also been more reluctant to restrict the personal liberties and freedoms of citizens.” We do not find, however, an inclusion of the value conflict between (health) security and (individual) freedoms in statistical models that analyze the variation of COVID-19 policies across democracies or autocracies. This value conflict is extensively discussed in legal studies (e.g., for Germany, see [12, 13]), political philosophy (e.g., [14, 15]), political psychology (e.g., [16, 17]), and economic history (e.g., [18]). From all these perspectives, this value conflict is considered to be relevant in times of pandemic. In Bilgen’s words, the question is “how a democratic order can overcome the security crisis while preserving its fundamental principles, such as individual freedom as a highest value” [13, p. 371]. Then, concepts (“securitas libertatis” [13]) and principles (“SAFE principles” [16]) are developed to guide preservation of supremacy, preponderance or primacy of one of both values or to restore the balance between freedom and security. Delanty [14] argues, that philosophical theories, such as utilitarianism and libertarianism, and ideas from philosophers such as Kant, Foucault, Agamben and Zizek, as well as nudge theory have shaped COVID-19 policies and draw attention to the problem of liberty or biopolitical securitization. He concludes that “the Coronavirus is more than a pathogen that threatens the lives of many people, but democracy is also in danger from the recent experiments with emergency government. … populations … have been disciplined in the late Foucauldian sense of the term to desire safety over liberty” [14, p. 14f.]. Vasilopoulos et al. [17] reveal the emotional mechanisms that lead citizens to decide to sacrifice their civil liberties in the light of threatened health security. We therefore claim that value preferences for freedom *and* security should be considered in explanations of variations of COVID-19 policies across jurisdictions. We find this value conflict also in the society, e.g., people were discussing the pros and cons in this value conflict in the social media. Political decision makers respond to value preferences in the society [19–21], we argue, however in different degrees. Since policy choices cannot simultaneously incorporate the totality of societal values, they are necessarily the selective result of competitive processes, favoring certain values over others, and excluding some values from the discourse altogether [20]. Distinguishing democracies and authoritarian regimes, our analyses show based on data for 40 countries that the variation in the stringency of COVID-19 policies in autocracies can be explained by value preferences of the population in this value conflict between freedom and security. In democracies, however, we do not find such a relationship. Governments in democratic political systems, we argue, are responsive to their constitutions and face prosecution by the judiciary if they violate the law or provisions of the constitution, limiting their capacity to implement strong COVID-19 policies. Nevertheless, their COVID-19 policies restricted citizens’ freedoms and liberties, which means that these policies were rather not responsive to citizens’ general preferences for freedom, democratic rights and liberties. By highlighting how autocracies respond to their citizens’ value preferences for security, this paper contributes to a better understanding of how autocracies might gain legitimacy in times of crises. Our study therefore provides quantitative support for the hypothesis that the Corona crisis will possibly strengthen autocratic regimes and weaken democratic political systems as the democracies fail to satisfy citizens’ demands for freedom while the autocracies respond to citizen’s demand for security.

The paper is structured as follows. Section 2 elaborates on the theories and previous findings that aim to explain variations in state COVID-19 responses–the relevance of values in politics, the differences found for democracies and authoritarian regimes, the relevance of policy diffusion as well as economic and health factors. In section 3, we explain material and methods. In section 4, we present our results. There are no substantial differences in the average stringency of COVID-19 policies over time between democracies and autocracies. Only the random effects models estimated for autocracies show a negative, significant association between value preferences of the population and the stringency of COVID-19 policies. In the conclusion, we argue that the Corona crisis provides an opportunity for autocracies to respond to value preferences of the population in a value conflict and thereby might gain legitimacy in times of crises.

## 2 Theories and previous findings

### Values and politics

Engler et al. [4, p. 2] as well as many others who research into the COVID-19 policies argue that governments are confronted with the “dilemma of weighing public health goals against democratic norms, rights and freedoms” resp. handling the trade-off between public health responses and democratic principles. We argue that this dilemma reflects a classical value conflict–the conflict between security and freedom, here between health security [22] and individual freedoms. In the context of the Corona crisis, health security and individual freedoms can be considered to be political values, not just individual values. Political values are correlated with attitudes towards policy goals [23–25], preferences for political parties [26], and electoral behavior [27–29]. We therefore argue that for political elites, it is rational to consider the value preferences in the society when adopting measures that, during normal times, either contradict fundamental democratic principles, such as freedom of movement or freedom of assembly, or are extraordinary anyway, e.g., the so-called “social distancing”. Toshkov et al. [3] distinguish two theoretical perspectives: (1) Party-political ideologies are related to the commitment to particular social values. COVID-19 policies are earlier or later based on these commitments. In particular, parties emphasizing traditional, authoritarian and nationalist values in their ideology, adopt faster and more restrictive COVID-19 policies. (2) Societal values and the overall freedom of the country influence COVID-19 policies. In more free societies, there is an appreciation of personal and collective liberties and freedoms–they are “protected by civil society” [3, p. 10] which imposes higher thresholds for governments to justify and enforce restrictions on fundamental freedoms. It is this latter theoretical perspective, which we investigate in this study, but we look at it from two sides and consider two societal values–freedom and security. We look at the trade-off between (health) security and (individual) freedoms considering not just the single values [30] but the value conflict. Stewart [20] delineates six forms of managing value conflict used by political and bureaucratic executives: structural separation, hybridization, casuistry, incrementalism, bias, and cycling.

### Authoritarian versus democratic systems

We claim that democracies and authoritarian regimes respond differently to this societal value conflict. In democracies, politicians should hesitate to limit individual liberties because they are inherent to democracy [31], and because negative public perceptions of restrictions of personal freedoms may jeopardize their reelection [32]. Differences in COVID-19 policies between authoritarian and democratic political systems were found by several empirical studies. In particular, democracies were slower in adapting the new policies [5, 6] or could possibly have been so [33]. The stronger the democratic institutions, the slower has the reaction in OECD countries been [2]. Autocracies have been more strict in their mobility restriction and contact tracing policies [8]. In democracies, the constitutional protection of individual liberties had a negative effect on restrictions of freedoms [4]. In democracies, political elites know that they are accountable [34–36]. Once the pandemic is over, or even before, other political as well as societal actors may question the legitimacy and proportionality of the COVID-19 policies and hold decision-makers accountable for their decisions. E.g., Maor & Howlett [1, p. 236] quote Israeli Minister of Finance Moshe Kahlon who argued that “surely there will be a national committee of inquiry, and no one wants [to have] to explain why there are Israeli corpses”. Such as Engler et al. [4], we argue that the strength of democratic institutions influences how political decision-makers in democracies handle the trade-off between health security and individual freedoms. The willingness to constrain civil liberties decreases with the degree to which democratic norms, rights and freedoms are protected and respected in non-pandemic times or times without a major crisis. The legal framework of democracies (rule of law; [37, 38]), and here we go beyond Engler et al. [4], establishes a point of reference for decision-makers which outweighs the value preferences in the society. Created as a safeguard against the misuse of power by governments [39], strong democratic institutions also establish an impediment or check to rely and respond directly to moods, attitudes or value preferences in the society [40, 41].

### Policy diffusion

In the name of health security, not only individual freedoms were constrained in an unprecedented way in many countries. Also, other measures implemented were very similar, although with some variation, hinting to politics of policy diffusion. Note that some measures, such as the closure of schools, are laid out in national pandemic plans, while others are not, e.g., the closure of borders, which means that the enforcement of the former cannot easily be interpreted as an indicator of policy diffusion. Policy diffusion has been defined as a process where policies in one political unit influence the politics of other units [42]. Already Meyer and Rowan [43] have argued that policies can spread because policy makers aim to conform to dominant international norms, in the Corona crisis to the recommendations of the World Health Organization (WHO), while considering also domestic constraints which results in a legislation that locally adapts policies to serve a domestic constituency. Four mechanisms are proposed to explain policy diffusion: learning, competition, coercion and emulation. Gilardi & Wasserfallen [42] derive a dominant stylized model of policy diffusion from the relevant literature in international relations and studies in federalism and argue that this model is based on the assumption that decisions are the result of fact-based assessments. They claim that instead policy diffusion should more be seen as a political process. E.g., diffusion may also involve unsuccessful policies, may be based on ideologically biased learning, may be shaped at the issue-definition stage or based on empirically false assumptions. In this framework, policy diffusion during the Corona crisis can considered to be based on the mechanism of emulation. Emulation scholars emphasize the social construction of appropriate policies [44], and that international organizations, such as the WHO, not only construct the norms fostering the appropriateness of policies but also promote policy diffusion [45, 46]. Such policies may be considered as legitimate [47]. However, in many cases, it is quite difficult to distinguish learning or coercion from emulation. E.g., in the Corona crisis, countries may also learn from mistakes of neighbouring countries’ COVID-19 policies; or announcements of the WHO together with announced financial support by other international or transnational organizations rather coerce some poorer countries to implement the proposed policies. Recently, Blatter et al. [48] have presented an alternative typology of policy diffusion, based on four motivational mechanisms (called interest-, rights-, ideology-, and recognition-driven policy diffusion) in an effort to overcome inconsistent operationalizations in empirical diffusion studies shown by Maggetti & Gilardi [49]. However, this typology is not yet suited to study diffusion processes in autocracies. In their empirical study on the first wave of the COVID-19 pandemic Rausis & Hoffmeyer‐Zlotnik [50] found tentative evidence for processes of policy diffusion in the field of mobility restrictions in Europe (EU, EFTA and UK). Engler et al. [4] and Sebhatu et al. [2] found diffusion effects for COVID-19 policies in European democracies and OECD countries, respectively.

### Economic and health factors

While diffusion effects may be present, we expect that the stringency of COVID-19 policies adopted by governments generally follow the epidemiological situation. The number of cases, deaths or case-fatality rates associated with COVID-19 vary strongly across countries and over time within countries [51]. For example, the case-fatality rates in June 2020 differed substantially between South Korea and Italy, while those of Germany and the United States were in between [52]. Hale et al. [53] show for the first phase of the pandemic that policies broadly track the reported COVID-19 cases. Thus, the varying severity of the COVID-19 situation could explain differences in the stringency of measures. Number of cases and deaths are only rough indicators for a country’s actual epidemiological situation: Number of confirmed cases are underestimates of actual cases and depend on testing policy, while number of deaths depend on how each country records and defines COVID-19 deaths. However, this is the only data available policy-makers can base their decisions on.

Apart from the epidemiological situation itself, a country’s resources and its vulnerability to the pandemic might influence the introduction of stringent measures as well. Countries with a low health care system capacity and a high proportion of risk groups for COVID-19, e.g. a large share of *elderly population*, are in need to introduce strict measures in order to prevent an overload of the health care system. We also assume that countries with a high GDP are able to introduce more stringent measures than countries with a low GDP, because they have more resources to react and are more capable of enduring damaging impacts on the economy due to lockdown measures than countries with a low GDP.

Number of deaths due to the virus, number of cases of people infected (epidemiological situation), share of risk groups, health care system capacity and GDP are common control variables in existing studies on the variation of COVID-19 measures [2, 4, 5, 11]. While the epidemiological situation and the share of risk groups should only influence the stringency of COVID-19 measures, we argue that the GDP and health care system capacity influence value preferences of the population towards freedom or security as well. A country’s GDP and health care system capacity are indicators of a country’s level of socioeconomic development, which leads to rising levels of existential security. According to the “revised theory of modernization”, *existential security* results in a shift from emphasis on survival values to emphasis on self-expression values [54]. Inglehart and Welzel [54] particularly mention the link between GDP and self-expression values.

## 3 Material and methods

### Data and sample

The analytic sample consists of 40 countries (20 democracies, 20 autocracies) for which information about the value conflict between freedom and security in the population is available in the World Values Survey (WVS) 2017–2020 dataset [55]. S1 Table provides a list of all countries included in the analysis. We excluded four countries (Iran, Myanmar, Ukraine, Zimbabwe) where the fieldwork of the WVS 2017–2020 continued until after March 11, 2020, when the WHO had already declared the COVID*-*19 outbreak a global pandemic. It is possible that the spread of COVID-19 and first political measures influenced the responses to the question about the value conflict between freedom versus security. In addition, Taiwan was excluded because of its unique trajectory of COVID-19 cases and policies [56]. Respondents in the WVS were asked the following question: “Most people consider both freedom and security to be important, but if you had to choose between them, which one would you consider more important?” We aggregated this information to the percentage of the population that considers freedom more important than security.

The stringency of policy responses were measured by the Stringency Index provided by the Oxford COVID-19 Government Response Tracker (OxCGRT) dataset (version July 2021; [57]). This index allows us to track COVID- 19 policy changes; starting from January 1, 2020, it has been measured daily. Varying between 0 and 100 it shows the stringency of lockdown policies, such as school and workplace closing, cancelation of public events and movement restrictions. S2 Table provides an overview of all nine indicators of the stringency index. All indicators have an ordinal scale of severity or stringency; the specific coding is shown in S2 Table as well. Detailed information about the calculation of the index and its indicators can be found in Hale et al. [57].

### Analytic strategy

We estimated random effects models: The models are linear panel regression models (daily observations nested in countries) with random effects (random intercept) at the country level. The models were estimated jointly for all 40 countries as well as separately for democracies (20 countries) and autocracies (20 countries). We distinguish democracies and autocracies using the Regimes of the World typology by Lührmann, Tannenberg, and Lindberg [58], which is included in the data provided by the Varieties of Democracy (V-Dem) Project [59, 60]. The analysis was restricted to the time span from February 01 to June 30, 2020, since we are interested in policy reactions during the first wave of the pandemic. We adopted this investigation period from Engler et al. [4]. In this wave, all countries had limited knowledge about adequate responses to COVID-19. For a robustness check, we estimated all models with different time spans by varying the end date (May 15, 2020; May 30, 2020; June 15, 2020; July 30, 2020) and the results were very similar.

The main models only controlled for variables that we expect to have an effect on both the Stringency Index and on the societal value conflict between freedom and security (confounders) to avoid overcontrol bias [61, 62]: *(1) gross domestic product (GDP*, *logged)*; health care system capacity, measured by the *(2) number of hospital beds per 1000 people* and *(3) share of the GDP spent on health care expenditures*; *(4) democracy level*. We used the data of the World Bank for the GDP (year 2019) and health care expenditures (year 2017), which is included in the World Value Survey 2017–2020 dataset [55]. The number of hospital beds per 1000 people (most recent year available) was from the OWID COVID-19 data [51] and the democracy level was measured by the Liberal Democracy Index of the V-Dem Institute (multiplied by 100; [59, 60]). GDP and health care system capacity is expected to influence not only the Stringency Index, but also the societal value conflict between freedom and security [54] (see section “Economic and health factors”). Likewise, the democracy level should not only be relevant for the stringency of COVID-19 policies (see section “Authoritarian versus democratic systems”), but is also likely to influence self-expression values within a population: “According to ‘institutional learning theory’, individuals’ values, preferences and behavior are heavily influenced by the institutional environments within which they operate” [63].

Main model with GDP, health care system capacity and democracy level as control variables:

$${Stringency\;Index}_{it}=\alpha +{freedom\;vs.\;security}_{i}+{GDP}_{i}+{hospital\;beds\;per\;1000\;people}_{i}+{health\;care\;expenditures}_{i}+{democracy\;level}_{i}+c_{i}+u_{it}$$

The indices i and t denote countries and date, respectively. The unobserved time-constant effect is called *c*_*i*_ and *u*_*it*_ denotes the time-varying error term.

In addition, we present models that further controlled for five variables that we expect to have an effect solely on the Stringency Index. First, to capture the severity of the COVID-19 situation, we included the *(1) daily number of new confirmed COVID-19 cases per million* (smoothed, 7-day rolling average; [51]). As a robustness check we used the daily number of new confirmed COVID-19 deaths per million (smoothed, 7-day rolling average; [51]) instead and the results were similar. Second, to account for diffusion effects, the models controlled for the *(2) worldwide daily average of the Stringency Index* (calculated from the OxCGRT data). Third, the models included the proportion of three COVID-19 risk groups. Those are the *(3) percentage of the total population aged over 60 years* in year 2015 [64], the *(4) percentage of people aged 20–79 who have type 1 or type 2 diabetes* in year 2019 [65], and the *(5) obesity prevalence* (BMI ≥ 30, crude rates) among adults aged over 20 years in year 2016 [66].

Model with additional control variables:

$${Stringency\;Index}_{it}=\beta_{0}+{freedom\;vs.\;security}_{i}+{GDP}_{i}+{hospital\;beds\;per\;1000\;people}_{i}+{health\;care\;expenditures}_{i}+{democracy\;level}_{i}+{infection\;cases\;per\;million}_{it}+{average\;Stringency\;Index}_{it}+{old\;population}_{i}+{diabetes\;prevalence}_{i}+{obesity\;prevalence}_{i}+c_{i}+u_{it}$$

Our variable of interest, freedom vs. security, is statistically significant correlated with all control variables, except worldwide average Stringency Index and diabetes prevalence (Pearson’s r, see S3 Table). However, none of these correlations is higher than 0.5, so there should be no collinearity between freedom vs. security and any of the control variables.

## 4 Results

### Descriptive statistics

Table 1 presents descriptive statistics for all variables included in the analysis in the three analytic samples (all countries, democracies, autocracies). There are no substantial differences in the average Stringency Index over time between democracies and autocracies. The average percentage of the population that considers freedom more important than security is higher in democracies (33.5%) than in autocracies (26.8%). However, as the range shows, there is a high variation between countries within both regime types. For the distribution of the societal value conflict and its single values for each country see S1 Fig and S1 Table, respectively.

**Table 1:** Descriptive statistics for all variables included in the analysis.

	All countries				Democracies				Autocracies			
	N. of time points	Mean	Std. Dev.	Range	N. of time points	Mean	Std. Dev.	Range	N. of time points	Mean	Std. Dev.	Range
*Time-variant variables*												
*Dependent variable*												
Stringency Index	6,040	56.87	32.62	0.00;100.00	3,020	56.32	32.20	0.00;100.00	3,020	57.41	33.03	0.00;100.00
*Control variables*												
Infection cases per million	4,918	14.19	34.36	-19.21;351.92	2,498	20.51	45.22	-19.21;351.92	2,420	7.66	14.30	0;87.68
Average stringency index	6,040	53.28	27.65	6.02;79.65	3,020	53.28	27.65	6.02;79.65	3,020	53.28	27.65	6.02;79.65
	N. of countries	Mean	Std. Dev.	Range	N. of countries	Mean	Std. Dev.	Range	N. of countries	Mean	Std. Dev.	Range
*Time-constant variables*												
*Explanatory variable*												
Freedom vs. security	40	30.86	14.35	4.20;70.94	20	33.61	15.91	4.20;70.94	20	28.12	12.40	6.64;55.66
*Control variables*												
GDP (logged)	40	9.66	0.82	7.75;11.09	20	10.05	0.70	8.58;11.09	20	9.28	0.75	7.75;10.29
Hospital beds per 1000 people	40	3.31	2.99	0.30;13.05	20	3.85	3.59	0.50;13.05	20	2.77	2.19	0.30;8.05
Health care expenditures	40	6.63	2.88	2.27;17.06	20	7.92	3.10	2.99;17.06	20	5.34	1.97	2.27;8.65
Liberal Democracy Index	40	40.11	26.24	4.30;84.60	20	62.81	15.96	35.90;84.60	20	17.41	8.57	4.30;29.10
Old population (% of population)	40	13.06	7.12	4.47;32.80	20	16.14	7.56	4.47;32.80	20	9.97	5.18	4.85;24.29
Diabetes prevalence	40	8.60	3.60	3.10;19.90	20	7.60	2.51	3.10;13.50	20	9.60	4.26	4.30;19.90
Obesity prevalence	40	19.71	10.12	2.10;37.30	20	21.93	9.37	4.40;37.30	20	17.50	10.59	2.10;33.40

**Fig 1:**
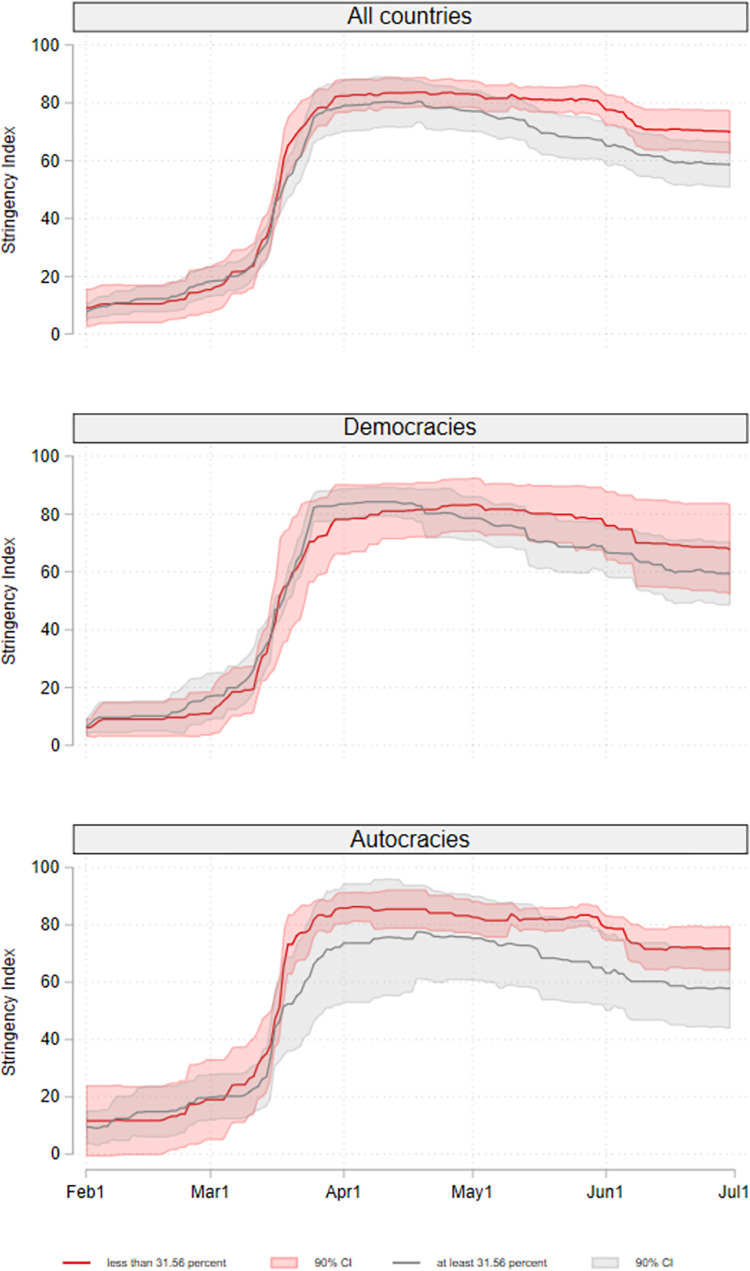
Stringency Index between February 01 to June 30, 2020. For countries with a high individual appreciation of freedom versus security and countries with a low individual appreciation of freedom versus security (median split: high = at least 31.56%, low = less than 31.56%); for all countries, and separately for democracies and autocracies. *Notes*: All countries: high: n = 20, low: n = 20; Democracies: high: n = 11, low: n = 9; Autocracies: high: n = 9, low: n = 11.

Fig 1 shows the development of the Stringency Index of all countries, democracies, and autocracies separately for countries with a high individual appreciation of freedom versus security and countries with a low individual appreciation of freedom versus security. High appreciation and low appreciation at this point mean that at least or less than 28% of the population consider freedom more important than security, respectively (median split). For autocracies, we find that except for a short period in February, the level of the Stringency Index between February 01 and June 30, 2020 of countries with a high appreciation of freedom versus security is consistently below that of countries with a low appreciation of freedom versus security. For democracies, we do not see this pattern until the end of April 2020. Within democracies, the period between end of March and mid of April 2020 shows an interesting pattern. Democratic countries with a high and low individual appreciation of freedom vs. security have similar levels of the Stringency Index in the beginning. However, at the end of March 2020 the stringency of lockdown measures in democratic countries with a high individual appreciation of freedom vs. security increases to a higher value than that of countries with a low individual appreciation of freedom vs. security and it remains at this level until mid of April 2020. It seems that countries with a high individual appreciation of freedom vs. security overreacted at the very beginning of the pandemic and relaxed their lockdown levels to a level below that of countries with a low individual appreciation of freedom vs. security only at a later point in time. However, it is important to note that the 90% confidence intervals of the lines overlap in all three samples, which means that there is no clear evidence of a difference between the two groups of countries. Moreover, from these descriptive results it is not possible to conclude that such a difference in the stringency of COVID-19 policies would be in fact due to value preferences in the population. The analyses in the next section will account for important confounders of this relationship.

### Impact of value preferences of the population on the stringency of COVID-19 policies

**Fig 2:**
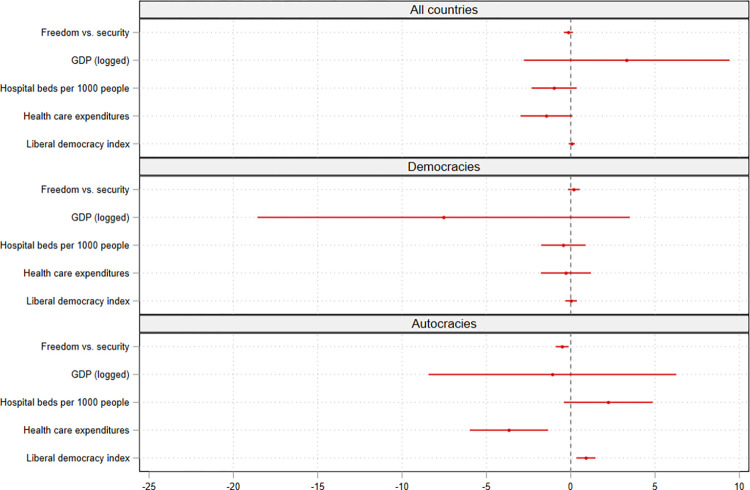
Random effects models for the Stringency Index. Notes: Coefficients are statistically significant if the 95% confidence intervals do not cover zero. Full regression tables are shown in Tables 2 and 3 (model 5).

Fig 2 shows the results of the main models that only controlled for variables that we expect to have an effect on both the Stringency Index and on the societal value conflict between freedom and security (estimated for all countries and separately for democracies and autocracies). There is no significant association between value preferences of the population and the Stringency Index in the sample that includes all countries. The coefficient is small and negative. In the sample that includes only democracies, we find a small positive, not significant association. The results of the third model, which was estimated only for autocracies, show a negative, significant association between value preferences of the population and the Stringency Index (p < 0.05). On average, if the percentage of the population that considers freedom more important than security increases by 1.0, the Stringency Index decreases by 0.5, ceteris paribus.

For the full regression tables see Tables 2 and 3. Model 5 is equivalent to the results shown in Fig 2 and model 6 additionally includes variables that that we expect to have an effect solely on the Stringency Index. Model 1 presents the bivariate relationship without control variables, as suggested by Lenz and Sahn [62]. To show how the effect size of the societal value conflict between freedom and security changes after including important confounders, the models 2, 3, and 4 only control for GDP, health care system capacity, and democracy level, respectively. Tables 2 and 3 also report two measures of goodness-of-fit, the adjusted overall R-squared and the Bayesian information criterion (BIC). In all three samples (all countries, democracies, autocracies), the goodness-of-fit is highest in model 6, which includes all control variables. However, because our aim is to explore the role of societal value preferences towards freedom or security for COVID-19 policies and not to fully explain the national variation of the stringency index, we do not interpret these measures of goodness-of fit further.

**Table 2:** Random effects models for the Stringency Index, all countries (n = 40).

	(1)	(2)	(3)	(4)	(5)	(6)
Freedom vs. security	-0.20	-0.21	-0.10	-0.21	-0.14	-0.12
	(0.12)	(0.13)	(0.14)	(0.13)	(0.14)	(0.18)
GDP (logged)		0.46			3.31	3.93
		(2.31)			(3.11)	(5.76)
Hospital beds per 1000 people			-0.89		-1.44^+^	-0.26
			(0.70)		(0.78)	(1.19)
Health care expenditures			-0.51		-0.99	-1.69
			(0.60)		(0.68)	(1.16)
Liberal Democracy Index				0.01	0.06	0.09
				(0.07)	(0.09)	(0.13)
Infection cases per million						0.10***
						(0.01)
Average Stringency Index						0.81***
						(0.01)
Old population (% of population)						-0.70
						(0.67)
Diabetes prevalence						-0.66
						(0.69)
Obesity prevalence						0.08
						(0.32)
Constant	62.96***	58.77**	67.39***	62.69***	39.42	3.09
	(4.09)	(21.21)	(4.77)	(4.37)	(26.67)	(46.06)
Adj. overall R^2^	0.01	0.01	0.02	0.01	0.02	0.58
BIC	59,218.0	59,226.0	59,183.5	59,226.0	59,154.7	41,773.3
Number of countries	40	40	40	40	40	40
Number of observations	6,040	6,040	6,040	6,040	6,040	4,918

Standard errors in parentheses

*** *p* < 0.001

** *p* < 0.01

* *p* < 0.05

^+^ *p* < 0.1

**Table 3:** Random effects models for the Stringency Index, democracies (n = 20) and autocracies (n = 20).

	Democracies						Autocracies					
	(1)	(2)	(3)	(4)	(5)	(6)	(1)	(2)	(3)	(4)	(5)	(6)
Freedom vs. security	-0.09	0.22^+^	0.02	0.06	0.18	0.09	-0.38	-0.35	-0.39^+^	-0.44*	-0.51**	-0.76**
	(0.12)	(0.13)	(0.14)	(0.13)	(0.18)	(0.28)	(0.24)	(0.24)	(0.22)	(0.22)	(0.19)	(0.29)
GDP (logged)		-9.93***			-7.53	-6.97		3.56			-1.08	-6.02
		(3.01)			(5.63)	(10.36)		(4.06)			(3.75)	(8.24)
Hospital beds per 1000 people			-0.55		-0.29	1.54			-3.18*		-3.67**	2.37
			(0.74)		(0.76)	(1.46)			(1.42)		(1.18)	(2.07)
Health care expenditures			-1.12*		-0.44	-0.78			1.04		2.22^+^	-4.20*
			(0.48)		(0.67)	(1.06)			(1.29)		(1.34)	(2.08)
Liberal Democracy Index				-0.26*	0.02	-0.06				0.69*	0.90**	1.25**
				(0.13)	(0.17)	(0.26)				(0.32)	(0.29)	(0.39)
Infection cases per million						0.09***						0.15***
						(0.01)						(0.02)
Average Stringency Index						0.80***						0.82***
						(0.01)						(0.01)
Old population (% of population)						-0.84						0.67
						(0.80)						(1.07)
Diabetes prevalence						-0.47						-0.41
						(1.29)						(0.87)
Obesity prevalence						0.63						-0.11
						(0.50)						(0.45)
Constant	59.21***	148.74***	64.44***	70.77***	128.52**	90.28	68.11***	34.30	82.53***	57.82***	79.46*	86.08
	(4.37)	(27.32)	(4.61)	(7.05)	(45.96)	(79.97)	(7.38)	(39.31)	(10.09)	(8.24)	(35.00)	(70.32)
Adj. overall R^2^	0.00	0.02	0.02	0.01	0.02	0.68	0.02	0.02	0.05	0.05	0.10	0.65
BIC	29,558.2	29,496.6	29,516.5	29,532.4	29,516.2	20,591.7	29,658.2	29,647.5	29,567.1	29,571.5	29,436.3	20,171.8
Number of countries	20	20	20	20	20	20	20	20	20	20	20	20
Number of observations	3,020	3,020	3,020	3,020	3,020	2,498	3,020	3,020	3,020	3,020	3,020	2,420

Standard errors in parentheses

*** *p* < 0.001

** *p* < 0.01

* *p* < 0.05

^+^ *p* < 0.1

As an additional analysis, we estimated models for all countries that included an interaction between the societal value conflict and the regime type (0 = Autocracy, 1 = Democracy; see S4 Table) instead of the separate analysis for democracies and autocracies. The interaction term is not statistically significant and we find a negative effect of the societal value conflict on the Stringency Index for autocracies (-0.36) and a positive effect that is close to zero for democracies (-0.36 + 0.41 = 0.05) in model 6, which includes all relevant control variables. However, it is important to note that our research question is not if the effect of the societal value conflict *differs* between democracies and autocracies, but if there is an effect within democracies and within autocracies. Our split sample analysis showed a small and not statistically significant positive effect of value preferences in the society on the stringency of COVID-19 policies in democracies, but a negative and statistically significant effect in autocracies. Therefore, it seems that there is a negative effect of the societal value conflict between freedom and security on the stringency of COVID-19 policies in autocracies, but not in democracies.

### Limitations

One main limitation of our analysis is the limited number of countries under study. We were only able to include countries in the analytic sample for which information about the societal value conflict between freedom and security is available in the WVS 2017–2020 dataset [55]. Due to the small sample size, our results might be sensible to the samples of countries. However, as a robustness check, we estimated the models excluding different single countries and the results were robust.

Moreover, our analysis is based on the assumption that the measured societal value conflict between freedom and security in the WVS is comparable across different countries. Alemán and Woods [67] questioned the measurement validity of survival–self-expression values. With respect to freedom and security which is relevant in this study, we might e.g., find self-censorship as well as a social desirability effect in favor of security as a consequence of autocratic indoctrination. However, Inglehart and Welzel and their co-authors have shown in numerous publications that measured values from the WVS are strongly linked to an extremely large number of social indicators—such as prosperity, equality, or democracy—which supports the cross-country comparability [68, 69].

One advantage of the random effects model is that it accounts for within- and between-country variation. However, it is not possible to identify a causal effect of the societal value conflict between freedom and security in the population on the stringency of COVID-19 measures, because there could still be unobserved heterogeneity that our analysis cannot account for. Although we attempted to account for all relevant observable confounders, we cannot completely eliminate the possibility that the assumption of exogeneity of both time-constant and time-varying unobserved heterogeneity may be violated.

## 5 Conclusion and discussion

The results indicated a negative association between the societal value conflict “freedom versus security” and the stringency of lockdown policies in autocracies, but not in democracies. This supports our anticipation that during the COVID-19 pandemic governments in democratic political systems are less responsive to their citizens’ value preferences for freedom, democratic rights and liberties than governments in autocratic political systems to their citizens’ value preference for security.

Our findings highlight how the Corona crisis provides an opportunity for autocracies to respond to value preferences of the population in a value conflict and thereby might gain legitimacy in times of crises. Recent studies on regime legitimation strategies in autocracies [70–73] emphasize specific support (first proposed by Easton [74]) as a source of legitimation. It is based on the regime’s claim to successfully satisfy citizens’ demands. Therefore, several authors subsume specific support to the type of performance-based legitimacy (e.g., [70, 75]). Instead, Gerschewski [71] argues that support should be considered to be a superset of legitimacy. His semantic analysis reveals that support also subsumes actors who hold anti-regime beliefs. These actors then behave as if they were in favor of the autocratic regime. In addition, we can also argue that there is an element of a democratic-procedural legitimation. Dukalskis & Gerschewski [73] distinguish two dimensions of democratic-procedural legitimation: (seemingly) rational elections, and responsiveness to the demands of the citizens. Seen from a theoretical perspective, autocracies have three advantages then: Their obvious capacity to respond to citizens’ demands for security strengthens their performance-based legitimacy, their democratic-procedural legitimation and the support by those citizens who actually hold anti-regime beliefs. Our study therefore provides quantitative support for the hypothesis that the Corona crisis will possibly strengthen autocratic regimes and weaken democratic political systems as the democracies fail to satisfy citizens’ demands for freedom while the autocracies respond to citizen’s demand for security (see also [76]). This hypothesis should be investigated by further studies. E.g., studies on the satisfaction with democracy indicate that only richer democracies might be affected and perceive a decline in citizens’ satisfaction with the way democracy works in their country [77].

The effort to managing value conflict between health security and individual freedoms may result in different dominant forms. Applying the delineation by Stewart [20] incrementalism and cycling may dominate in democracies because they have to deal with the value conflict beyond institutional limitations while a bias in favor of security may dominate in autocracies. This is not only a formal difference: incrementalist and oscillating policies are stressful for the citizens. They generate political emotions against democratic governments and their COVID-19 policies. Bias towards security, on the other hand, creates no additional stress in autocracies, which are in any case used to suppress any opposition. Future studies should investigate whether such a difference can be empirically found.

## Supporting information

S1 Fig Distribution of the percentage of the population that considers freedom more important than security. (TIF)

S1 Table List of countries and the percentage of the population that considers freedom more important than security. (DOCX)

S2 Table Indicators of the Stringency Index. (DOCX)

S3 Table Pairwise correlations. (DOCX)

S4 Table Random effects models for the Stringency Index with interaction term between regime type and freedom vs. security, all countries (n = 40). (DOCX)
