# Supplementary figures and images for: The value conflict between freedom and security: Explaining the variation of COVID-19 policies in democracies and autocracies
Source: PLoS One. 2022 Sep 9;17(9):e0274270. doi: 10.1371/journal.pone.0274270 (PMC9462556; doi:10.1371/journal.pone.0274270)

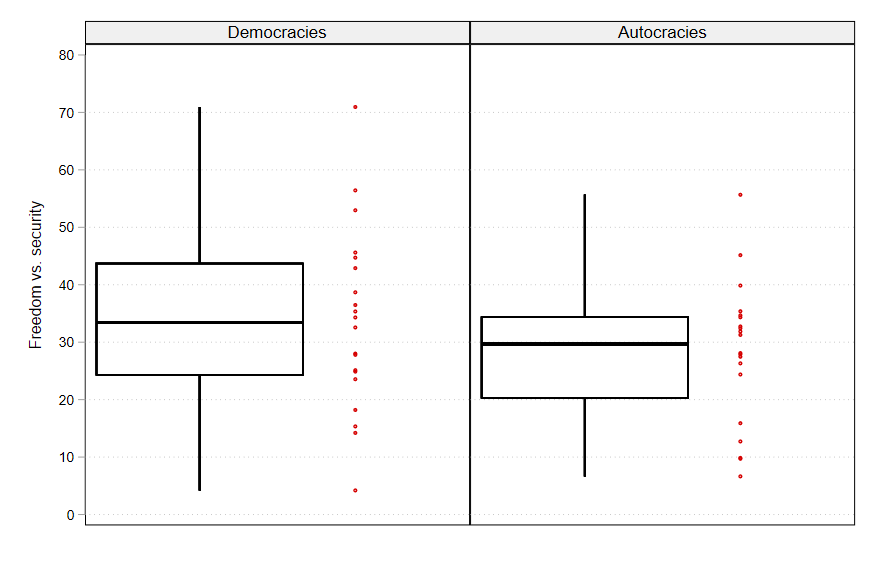

Supplement: S1 Fig — (TIF) [file pone.0274270.s001.tif]
