# Supplementary material for: The value conflict between freedom and security: Explaining the variation of COVID-19 policies in democracies and autocracies
Source: PLoS One. 2022 Sep 9;17(9):e0274270. doi: 10.1371/journal.pone.0274270 (PMC9462556; doi:10.1371/journal.pone.0274270)
Supplement: S1 Table — (DOCX) [file pone.0274270.s002.docx]

**Table S1. List of countries and the percentage of the population that considers freedom more important than security.**

| Democracies  (n=20) | |  | Autocracies  (n=20) | |
| --- | --- | --- | --- | --- |
| Country | Freedom vs. security |  | Country | Freedom vs. security |
| Argentina | 36.47 |  | Bangladesh | 27.47 |
| Australia | 56.43 |  | Bolivia | 15.90 |
| Brazil | 24.88 |  | China | **6.64** |
| Chile | 35.35 |  | Egypt | 12.72 |
| Colombia | 32.57 |  | Ethiopia | 32.74 |
| Cyprus | 38.68 |  | Iraq | 9.86 |
| Ecuador | 23.55 |  | Jordan | 9.71 |
| Germany | 44.72 |  | Kazahstan | 27.89 |
| Greece | 45.61 |  | Kyrgyzstan | 35.39 |
| Guatemala | 25.13 |  | Lebanon | 26.32 |
| Indonesia | **4.20** |  | Malaysia | 28.10 |
| Japan | 14.21 |  | Nicaragua | 34.67 |
| Mexico | 27.80 |  | Pakistan | 45.15 |
| New Zealand | 52.97 |  | Philippines | 31.86 |
| Nigeria | 28.01 |  | Russia | 24.38 |
| Peru | 15.34 |  | Serbia | **55.66** |
| Romania | 34.31 |  | Tajikistan | 34.33 |
| South Korea | 42.89 |  | Thailand | 31.27 |
| Tunisia | 18.20 |  | Turkey | 39.86 |
| United States | **70.94** |  | Vietnam | 32.42 |
