# Supplementary material for: The value conflict between freedom and security: Explaining the variation of COVID-19 policies in democracies and autocracies
Source: PLoS One. 2022 Sep 9;17(9):e0274270. doi: 10.1371/journal.pone.0274270 (PMC9462556; doi:10.1371/journal.pone.0274270)
Supplement: S2 Table — (DOCX) [file pone.0274270.s003.docx]

**Table S2. Indicators of the Stringency Index.**

| Indicator | Description | Coding |
| --- | --- | --- |
| School closure | Record closings of schools and universities | 0 – no measures  1 – recommend closing  2 – require closing (only some levels or categories)  3 – require closing (all levels) |
| Workplace closure | Record closings of workplaces | 0 – no measures  1 – recommend closing  2 – require closing (some sectors or categories)  3 – require closing (for all but essential workplaces) |
| Cancelation of public events | Record canceling public events | 0 – no measures  1 – recommend canceling  2 – require canceling |
| Restrictions on gatherings | Record limits on gatherings | 0 – no restrictions  1 – restrictions on very large gatherings (>1000 people)  2 – restrictions on gatherings between 101-1000 people  3 – restrictions on gatherings between 11-100 people  4 – restrictions on gatherings of 10 people or less |
| Closure of public transportation | Record closings of public transport | 0 – no measures  1 – recommend closing  2 – require closing |
| Stay at home requirements | Record orders to “shelter-in-place” and otherwise confined to the home | 0 – no measures  1 – recommend not leaving house  2 – require not leaving house with exceptions for daily  exercise, grocery shopping, and 'essential' trips  3 – require not leaving house with minimal  exceptions (e.g. allowed to leave once a week) |
| Restrictions on internal movements | Record restrictions on internal movement between cities/regions | 0 – no measures  1 – recommend not to travel between regions/cities  2 – internal movement restrictions in place |
| International travel controls | Record restrictions on international travel | 0 – no restrictions  1 – screening arrivals  2 – quarantine arrivals from some or all regions  3 – ban arrivals from some regions  4 – ban on all regions or total border closure |
| Public information campaigns | Record presence of public information | 0 – no COVID-19 public information campaign  1 – public officials urging caution about COVID-19  2 – coordinated public information campaign (e.g. across traditional and social media) |

Notes: The Stringency Index is calculated using these nine indicators. Each indicator has an additional weight of general scope corresponding to whether the policy has been applied locally or nationwide. Detailed information about the calculation of the index and its indicators can be found in Hale et al. (2021)

Source: Codebook for the Oxford Covid-19 Government Response Tracker (version 3.4), retrieved from https://github.com/OxCGRT/covid-policy-tracker/blob/master/documentation/codebook.md
