# Supplementary material for: The value conflict between freedom and security: Explaining the variation of COVID-19 policies in democracies and autocracies
Source: PLoS One. 2022 Sep 9;17(9):e0274270. doi: 10.1371/journal.pone.0274270 (PMC9462556; doi:10.1371/journal.pone.0274270)
Supplement: S3 Table — (DOCX) [file pone.0274270.s004.docx]

**Table S3. Pearson’s r correlations (pairwise).**

| Variables | 1 | 2 | 3 | 4 | 5 | 6 | 7 | 8 | 9 | 10 | 11 |
| --- | --- | --- | --- | --- | --- | --- | --- | --- | --- | --- | --- |
| 1 Stringency Index | 1 |  |  |  |  |  |  |  |  |  |  |
| 2 Freedom vs.  security | -0.09* | 1 |  |  |  |  |  |  |  |  |  |
| 3 GDP (logged) | -0.02 | 0.37* | 1 |  |  |  |  |  |  |  |  |
| 4 Hospital beds per 1000 people | -0.08* | 0.16* | 0.55* | 1 |  |  |  |  |  |  |  |
| 5 Health care expenditures | -0.11* | 0.48* | 0.51* | 0.32* | 1 |  |  |  |  |  |  |
| 6 Liberal Democracy Index | -0.02 | 0.37* | 0.64* | 0.32* | 0.59* | 1 |  |  |  |  |  |
| 7 Infection cases per million | 0.20* | 0.08* | 0.11* | -0.06* | 0.21* | 0.19* | 1 |  |  |  |  |
| 8 Average Stringency Index | 0.84* | 0.00 | 0.00 | -0.00 | 0.00 | -0.00 | 0.13* | 1 |  |  |  |
| 9 Old population | -0.08* | 0.41* | 0.79* | 0.72* | 0.55* | 0.60* | 0.05* | -0.00 | 1 |  |  |
| 10 Diabetes prevalence | -0.02 | -0.02 | -0.01 | -0.27* | -0.05* | -0.22* | 0.01 | -0.00 | -0.22* | 1 |  |
| 11 Obesity prevalence | -0.03* | 0.26* | 0.48* | -0.07* | 0.50* | 0.31* | 0.22* | -0.00 | 0.21* | 0.24* | 1 |

*Note:* * Correlation is significant at the 0.05 level (two-tailed)
