# Supplementary material for: The value conflict between freedom and security: Explaining the variation of COVID-19 policies in democracies and autocracies
Source: PLoS One. 2022 Sep 9;17(9):e0274270. doi: 10.1371/journal.pone.0274270 (PMC9462556; doi:10.1371/journal.pone.0274270)
Supplement: S4 Table — (DOCX) [file pone.0274270.s005.docx]

**Table S4. Random effects models for the Stringency Index with interaction term between regime type and freedom vs. security, all countries (n=40)**

|  | (1) | (2) | (3) | (4) | (5) | (6) |
| --- | --- | --- | --- | --- | --- | --- |
| Freedom vs. security | -0.20 | -0.38^+^ | -0.39^+^ | -0.36^+^ | -0.38^+^ | -0.36^+^ |
|  | (0.12) | (0.20) | (0.21) | (0.19) | (0.20) | (0.20) |
| Regime type (ref. = autocracy) |  | -8.91 | -9.24 | -11.31 | -9.36 | -14.44 |
|  |  | (8.47) | (8.70) | (8.14) | (9.80) | (10.09) |
| Freedom vs. security*Regime type |  | 0.29 | 0.32 | 0.51^+^ | 0.29 | 0.41 |
|  |  | (0.26) | (0.28) | (0.26) | (0.27) | (0.29) |
| GDP (logged) |  |  | -0.68 |  |  | 1.35 |
|  |  |  | (2.83) |  |  | (3.36) |
| Hospital beds per 1000 people |  |  |  | -0.43 |  | -0.74 |
|  |  |  |  | (0.58) |  | (0.72) |
| Health care expenditures |  |  |  | -1.71^*^ |  | -1.81^*^ |
|  |  |  |  | (0.80) |  | (0.82) |
| Liberal Democracy Index |  |  |  |  | 0.02 | 13.23 |
|  |  |  |  |  | (0.16) | (16.82) |
| Constant | 62.96^***^ | 68.11^***^ | 74.56^**^ | 77.94^***^ | 67.88^***^ | 64.38^*^ |
|  | (4.09) | (6.16) | (27.58) | (7.08) | (6.69) | (31.61) |
|  |  |  |  |  |  |  |
| Adj. overall R^2^ | 0.01 | 0.01 | 0.01 | 0.03 | 0.01 | 0.03 |
| BIC | 59,218.0 | 59,212.7 | 59,220.4 | 59,132.9 | 59,221.3 | 59,134.5 |
| Number of countries | 40 | 40 | 40 | 40 | 40 | 40 |
| Number of observations | 6,040 | 6,040 | 6,040 | 6,040 | 6,040 | 6,040 |

Standard errors in parentheses

*** *p* < 0.001, ** *p* < 0.01, * *p* < 0.05, ^+^ *p* < 0.1
